# Effects of Exercise on Ocular Parameters and Oxidative Stress Biomarkers in Saliva and Tear Fluid of Horses

**DOI:** 10.1002/vms3.71153

**Published:** 2026-08-12

**Authors:** Ferda Turgut, Latif Emrah Yanmaz, Omer Tarik Orhun, Betul Apaydin Yildirim, Yakup Kocaman, Mumin Gokhan Senocak, Taner Arslan, Vildan Aslan Canatan

**Affiliations:** ^1^ Department of Surgery, Faculty of Veterinary Medicine Cukurova University Adana Turkey; ^2^ Department of Surgery, Faculty of Veterinary Medicine Burdur Mehmet Akif Ersoy University Burdur Turkey; ^3^ Department of Surgery, Faculty of Veterinary Medicine Necmettin Erbakan University Konya Turkey; ^4^ Department of Biochemistry, Faculty of Veterinary Medicine Ataturk University Erzurum Turkey; ^5^ Department of Surgery, Faculty of Veterinary Medicine Yozgat Bozok University Yozgat Turkey; ^6^ Department of Surgery, Faculty of Veterinary Medicine Ataturk University Erzurum Turkey

**Keywords:** antioxidants, exercise, eye pressure, non‐invasive, oxidative

## Abstract

**Background:**

Physical exercise is a physiological stressor in horses and may induce oxidative stress through increased production of reactive oxygen species. Saliva and tear fluid are accessible non‐invasive matrices that may reflect oxidative balance, while ocular parameters including intraocular pressure (IOP), tear secretion (TS) and central corneal thickness (CCT) are key components of ocular homeostasis.

**Objective:**

To evaluate the effects of a standardised submaximal exercise session on IOP, TS and CCT, and on oxidative stress biomarkers (CAT, SOD, GSH, MDA, IMA) measured in saliva and tear fluid.

**Method:**

Thirty‐six clinically healthy Arabian horses underwent a 30‐min submaximal field exercise (walk–trot–canter). Heart rate, ocular parameters (Schirmer tear test‐1, rebound tonometry, ultrasonic pachymetry) and saliva and tear fluid samples were collected immediately before and after exercise. Data normality was assessed using the Shapiro–Wilk test, and paired‐samples *t*‐tests or Wilcoxon signed‐rank tests were applied as appropriate.

**Result:**

Heart rate increased significantly after exercise (*p* < 0.001). IOP and CCT did not change significantly, whereas TS decreased modestly (*p* = 0.033). In saliva, CAT and SOD increased and MDA decreased (all *p* < 0.001), with no changes in GSH or IMA. In tear fluid, CAT, GSH and SOD increased, while IMA and MDA decreased significantly.

**Conclusion:**

Submaximal exercise elicited antioxidant‐dominant shifts in both saliva and tear fluid, while ocular parameters remained largely stable except for a mild reduction in TS. Tear fluid, alongside saliva, may provide complementary information on exercise‐associated physiological and oxidative responses in horses under routine training conditions.

## Graphical abstract

Submaximal exercise induced measurable oxidative responses in saliva and tear fluid of horses, while ocular parameters remained largely stable except for a mild reduction in tear secretion. Saliva and tear fluid may serve as practical, minimally invasive matrices for monitoring exercise‐associated physiological and oxidative responses in horses.

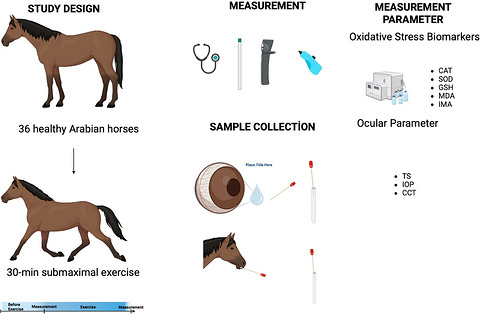

## 1 Introduction

Physical exercise is widely recognised as a natural physiological stressor that affects thermoregulation and homeostatic balance in horses through pronounced cardiovascular and metabolic responses (Seabra et al. 2019). Increased metabolic activity during exercise elevates heart rate, blood pressure and core body temperature, thereby activating thermoregulatory mechanisms to dissipate excess heat and limit exercise‐induced hyperthermia that may compromise health and performance (Seabra et al. 2019; Aragona et al. 2022). Exercise‐induced physiological stress is associated with increased production of reactive oxygen species due to enhanced oxygen consumption and mitochondrial activity, and oxidative stress occurs when antioxidant defence capacity is exceeded, potentially leading to oxidative damage of lipids, proteins and cellular structures (Powers and Jackson 2008; Halliwell and Gutteridge 2015).

Tear fluid and saliva are readily accessible biological matrices that reflect local and systemic oxidative balance and have been increasingly used as non‐invasive tools to detect oxidative stress (Wakamatsu et al. 2013; Maciejczyk et al. 2021). Tear fluid contains enzymatic and non‐enzymatic antioxidant systems that contribute to ocular surface integrity, and oxidative stress–related markers have been detected in tear samples, supporting its relevance in ocular physiology and stress assessment (Wakamatsu et al. 2013; Seen and Tong 2018). These characteristics make tear fluid a promising non‐invasive matrix for investigating redox alterations associated with physiological and pathological processes (Wakamatsu et al. 2013; Best et al. 2015; Yildirim and Yildirim 2023). Saliva serves as an important antioxidant barrier against free radicals and is widely recognised as a reliable non‐invasive medium for evaluating systemic oxidative stress and physiological stress responses (Maciejczyk et al. 2021; Yildirim and Yildirim 2023). The identification of reliable non‐invasive biomarkers may provide practical tools for monitoring training adaptation, physiological stress responses and welfare in horses under routine management and exercise conditions.

In horses, ocular parameters including intraocular pressure (IOP) and tear secretion (TS) are integral to ocular homeostasis and may be influenced by physiological stressors (Cinar et al. 2025). IOP reflects the balance between aqueous humour production and outflow, whereas TS contributes to ocular surface protection and stability (Orzalesi et al. 2000; Cinar et al. 2025). In addition to its influence on IOP measurements, CCT is an important indicator of corneal status and may be affected by physiological changes associated with exercise (Aragona et al. 2022). Exercise‐related changes in IOP have been reported in horses, suggesting that physical activity can induce measurable ocular responses, possibly through physiological adaptations associated with exercise, including changes in autonomic regulation and fluid balance, while structural factors, including central corneal thickness, may further affect the accuracy of IOP measurements (Zakrzewska et al. 2019; de Oliveira Bacchin et al. 2021; Aragona et al. 2022).

Previous studies have reported exercise‐associated changes in ocular parameters in horses and have demonstrated that saliva can be used as a non‐invasive matrix for monitoring physiological and oxidative responses to exercise (Yadav et al. 2018; Aragona et al. 2022; Hostovská et al. 2024). However, information regarding oxidative stress biomarkers in equine tear fluid following exercise is limited. This study aimed to evaluate the effects of physical exercise on ocular parameters, including IOP, TS and CCT, as well as on oxidative stress biomarkers measured in saliva and tear fluid of horses by comparing values obtained before and after exercise. We hypothesised that exercise would induce significant alterations in ocular physiological parameters and would be associated with measurable changes in oxidative stress biomarkers in both biological fluids.

## 2 Materials and Methods

Ethical approval for this study was obtained from the Atatürk University Animal Experiments Local Ethics Committee (Approval No. 2025/14‐343). The study was conducted at the Atatürk University Veterinary Faculty Animal Hospital, Erzurum, Türkiye.

### 2.1 Animals

Thirty‐six clinically healthy Arabian horses of both sexes were enrolled in the study, including 5 mares and 31 stallions, with a mean body weight of 410 ± 30 kg and an age range of 15 months to 15 years. Written informed owner consent was obtained for all animals prior to inclusion in the study. All horses were housed under identical management conditions and were fed high‐quality hay and mixed grain concentrate three times daily (06:00, 12:00 and 19:00), with free access to water. Prior to inclusion, each horse underwent a comprehensive clinical examination, complete ophthalmic assessment and routine haematological analyses to confirm health status. Horses showing any signs of ocular discomfort or systemic illness were excluded from the study.

### 2.2 Exercise Protocol

All exercise sessions were performed under the supervision of the same rider (groom/trainer) on a flat, obstacle‐free track. To minimise circadian and inter‐individual variability, all training sessions were conducted at 11:00 AM. The exercise protocol consisted of a standardised submaximal running session designed to simulate routine training conditions. The exercise intensity was not designed as maximal effort but reflected routine submaximal field exercise conditions. The total exercise duration was 30 min and included 10 min of walking for warm‐up, followed by 15 min of trotting at a constant submaximal speed and 5 min of cantering on a straight, level track. Jumping or obstacle‐related activities were not included. Heart rate was recorded immediately before exercise and immediately after completion of the exercise session.

### 2.3 Ocular Parameters

Physiological, ocular, saliva and tear fluid measurements were obtained immediately before exercise and immediately after completion of the exercise session. Ocular parameters were assessed first, followed by collection of saliva and tear fluid samples at each time point. All ocular examinations were performed by the same examiner throughout the study. All examinations were performed with horses standing calmly, with the head positioned above the level of the withers. TS was measured first using the Schirmer tear test‐1 without topical anaesthesia. A standard Schirmer tear test strip was placed in the lateral third of the lower conjunctival fornix of each eye for 1 min, and the examiner manually ensured eyelid opening during measurement.

IOP was measured following TS assessment using a rebound tonometer (Tonovet, Icare, Vantaa, Finland) set to the equine mode. No sedatives, local anaesthetic blocks or topical ocular anaesthesia were administered prior to tonometry. The upper eyelid was gently held without applying digital pressure to the globe. Six successive rebound measurements were obtained for each eye, and the mean IOP value displayed by the device was used for analysis. The order of right and left eye measurements was determined randomly. A new disposable probe was used for each animal. After completion of TS and IOP measurements, CCT was assessed using ultrasonic pachymetry (Ipac, Reichert, NY, USA). Topical anaesthesia was achieved by instillation of 0.5% proparacaine hydrochloride (Alcaine; Alcon‐Couvreur, Puurs, Belgium). After 1 min, the probe was positioned perpendicularly at the central cornea, and the mean of three consecutive measurements was recorded. Measurements were obtained from both eyes, and the mean value of the right and left eyes was used for statistical analysis.

### 2.4 Oxidative Stress Biomarkers

Saliva and tear fluid samples were collected from all horses at two predefined time points: immediately before exercise and immediately after the exercise session, following IOP measurements. Tear fluid samples were obtained using sterile cotton swabs (Deltalab, Barcelona, Spain) placed gently in the lower conjunctival fornix, while saliva samples were collected using sterile cotton swabs positioned in the oral cavity, avoiding feed contamination and excessive stimulation. All samples were collected with the horses in a calm standing position.

Immediately after collection, saliva and tear fluid samples were transferred into tubes containing 20 mM Tris–HCl buffer (pH 6.5) and incubated at +4°C for 30 min. Samples were then centrifuged at 10,000 rpm for 15 min, and the supernatants were aliquoted into sterile Eppendorf tubes and stored at −80°C until biochemical analysis.

Levels of malondialdehyde (MDA) (Yoshioka et al. 1979), ischemia‐modified albumin (IMA) (Bar‐Or et al. 2000), superoxide dismutase (SOD) (Sun et al. 1988), glutathione (GSH) (Tietze 1969) and catalase (CAT) (Góth 1991) were determined in saliva and tear fluid supernatants using a microplate reader (BioTek Quant MQX200 ELISA Reader, USA).

### 2.5 Data Analysis

The sample size was calculated with IOP as the primary outcome variable, using a paired‐samples *t*‐test to compare pre‐ and post‐exercise measurements. The significance level was set at 0.05 (two‐sided), with a target statistical power of 90%. Estimates of variability were based on previously published data in clinically healthy Arabian horses (Cinar et al. 2025), in which mean IOP values of 29.5 ± 7.2 mmHg and 34.2 ± 6.8 mmHg were reported under different environmental conditions. A moderate within‐subject correlation between repeated measurements was assumed (*r* = 0.50). Under these assumptions, a minimum of 24 horses was required to detect a mean difference of approximately 4.5 mmHg in IOP, corresponding to a clinically relevant moderate effect. To allow for potential inter‐individual variability and to ensure sufficient power for secondary outcomes, including TS, CCT and oxidative stress biomarkers, the final sample size was increased, and a total of 36 horses were included in the study.

All statistical analyses were performed using MedCalc Statistical Software (MedCalc Software Ltd., Ostend, Belgium). The normality of data distribution for each variable was assessed using the Shapiro–Wilk test. Comparisons between pre‐exercise and post‐exercise measurements were performed using paired‐samples *t*‐tests for IOP, TS, salivary MDA, IMA, SOD, CAT and tear fluid CAT. Wilcoxon signed‐rank tests were used for salivary GSH, tear fluid GSH, MDA, IMA, SOD and CCT. A *p*‐value of less than 0.05 was considered statistically significant. Normally distributed variables were expressed as mean ± standard deviation, whereas non‐normally distributed variables were presented as median and interquartile range (IQR).

## 3 Results

Heart rate increased significantly following exercise, rising from 53.89 ± 17.40 beats/min before exercise to 68.94 ± 18.36 beats/min after exercise (*p* < 0.001). IOP and CCT did not differ significantly following exercise, whereas TS values showed a modest but significant decrease after exercise (*p* = 0.033) (Table 1).

**TABLE 1:** Changes in ocular parameters before and after exercise.

Parameter	Before exercise	After exercise	*p* value
**IOP (mmHg)**	29.27 ± 6.80	27.89 ± 7.44	0.175
**TS**	21.76 ± 5.58	18.41 ± 5.24	0.033
**CCT (µm)**	854.50 (806.88–888.00)	849.50 (804.75–884.63)	0.060

*Note*: Data are presented as mean ± SD or median (interquartile range) according to data distribution.

Abbreviations: CCT, central corneal thickness; IOP, intraocular pressure; TS, tear secretion.

**FIGURE 1:**
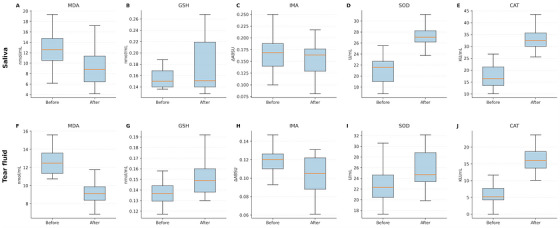
Boxplots showing pre‐ and post‐exercise values of oxidative stress biomarkers in saliva (A–E) and tear fluid (F–J). Salivary biomarkers included malondialdehyde (MDA), glutathione (GSH), ischemia‐modified albumin (IMA), superoxide dismutase (SOD) and catalase (CAT) (A–E). Corresponding biomarkers measured in tear fluid are presented in panels F–J. Boxes represent the interquartile range (IQR), horizontal lines indicate the median and whiskers represent the data range.

In saliva, CAT activity increased markedly after exercise (17.01 ± 5.28 to 32.54 ± 4.92 KU/mL, *p* < 0.001), and SOD activity also showed a significant increase (21.12 ± 2.56 to 27.10 ± 2.17 U/mL, *p* < 0.001), whereas MDA concentrations decreased significantly (12.52 ± 3.41 to 8.91 ± 3.42 nmol/mL, *p* < 0.001). No significant changes were detected in salivary GSH or IMA levels (Figure 1, Table 2).

**TABLE 2:** Changes in salivary and tear fluid oxidative stress biomarkers before and after exercise.

Parameter	Sample	Before exercise	After exercise	*p* value
**CAT (KU/mL)**	Saliva	17.01 ± 5.28	32.54 ± 4.92	< 0.001
**GSH (nmol/mL)**		0.15 (0.14–0.16)	0.15 (0.14–0.23)	0.13
**IMA (ΔABSU)**		0.17 ± 0.04	0.16 ± 0.03	0.21
**MDA (nmol/mL)**		12.52 ± 3.41	8.91 ± 3.42	< 0.001
**SOD (U/mL)**		21.12 ± 2.56	27.10 ± 2.17	< 0.001
**CAT (KU/mL)**	Tear fluid	6.00 ± 2.90	16.15 ± 3.63	< 0.001
**GSH (nmol/mL)**		0.14 (0.13–0.14)	0.15 (0.14–0.16)	0.007
**IMA (ΔABSU)**		0.12 (0.11–0.13)	0.11 (0.09–0.13)	0.039
**MDA (nmol/mL)**		12.48 (11.33–13.68)	9.10 (8.18–9.90)	< 0.001
**SOD (U/mL)**		22.31 (20.37–24.70)	24.71 (23.29–29.22)	0.009

*Note*: Data are presented as mean ± SD or median (interquartile range) according to data distribution.

Abbreviations: CAT, catalase; GSH, glutathione; IMA, ischemia‐modified albumin; MDA, malondialdehyde; SOD, superoxide dismutase.

In tear fluid, CAT activity increased from 6.00 ± 2.90 to 16.15 ± 3.63 KU/mL (*p* < 0.001), accompanied by significant increases in GSH (0.14 [0.13–0.14] to 0.15 [0.14–0.16] nmol/mL, *p* = 0.007) and SOD (22.31 [20.37–24.70] to 24.71 [23.29–29.22] U/mL, *p* = 0.009). Conversely, tear fluid IMA and MDA concentrations decreased significantly after exercise (IMA: 0.12 [0.11–0.13] to 0.11 [0.09–0.13] ΔABSU, *p* = 0.039; MDA: 12.48 [11.33–13.68] to 9.10 [8.18–9.90] nmol/mL, *p* < 0.001).

## 4 Discussion

The present study shows that submaximal exercise induces clear changes in oxidative stress biomarkers in horses, particularly in saliva and tear fluid, while exerting limited effects on ocular parameters. Exercise‐related stress affects autonomic regulation, fluid homeostasis and redox balance, which may be reflected not only in systemic biological fluids but also in ocular surface physiology (Seen and Tong 2018; Aragona et al. 2022). Importantly, exercise does not invariably result in detrimental oxidative stress, as moderate exercise may stimulate adaptive antioxidant responses through activation of endogenous antioxidant defence mechanisms, thereby promoting redox homeostasis (Ott et al. 2022). Exercise was associated with increased antioxidant enzyme activities and reduced lipid peroxidation in both biological fluids, with tear fluid demonstrating a more pronounced response. This pattern may reflect an adaptive antioxidant response rather than overt oxidative damage. Moderate or submaximal exercise can activate endogenous antioxidant defence mechanisms, a phenomenon consistent with exercise‐induced hormesis, in which a transient physiological challenge enhances redox regulation without necessarily resulting in measurable oxidative injury. The increases in CAT, SOD and GSH observed in the present study may therefore indicate enhanced antioxidant capacity in response to exercise. In addition, the decrease in MDA and IMA concentrations may suggest that the exercise intensity was insufficient to induce marked lipid peroxidation or ischemia‐related oxidative damage, particularly in horses accustomed to routine training (Radak et al. 2008; Gomez‐Cabrera et al. 2008; Powers and Jackson 2008). This interpretation is consistent with studies reporting adaptive antioxidant responses after moderate exercise, although it contrasts with reports in which more intense or prolonged exercise increased oxidative damage markers (Radak et al. 2008; Gomez‐Cabrera et al. 2008). Differences in exercise intensity, duration, training status, sampling time and biological matrix may explain these divergent findings. The timing of sample collection should also be considered when interpreting the oxidative stress findings. Because samples were obtained immediately after exercise, the antioxidant‐dominant profile observed in the present study may partly reflect the selected sampling time, and different responses might have been detected during later recovery periods (Ott et al. 2022).

In contrast, IOP and CCT remained unchanged, although TS decreased modestly after exercise. These findings support the hypothesis that physical exercise elicits measurable oxidative stress responses in saliva and tear fluid, while its effects on ocular physiological parameters are relatively subtle. These findings suggest that saliva and tear fluid might be suitable and minimally invasive matrices for monitoring exercise‐induced physiological and oxidative responses in horses. However, these biomarkers need to be interpreted along with clinical and physiological parameters and not in isolation.

The absence of significant changes in IOP and CCT is in agreement with observations in clinically healthy horses, where these parameters showed limited variability in the absence of ocular or systemic disease (Zisopoulou et al. 2023). In contrast, TS decreased modestly after exercise, suggesting a mild, short‐lived change at the ocular surface. Tear film function in horses is shaped not only by aqueous volume but also by physicochemical features, including osmolality and electrolyte composition, which support surface stability in clinically healthy animals (Best et al. 2015). In this context, a small post‐exercise reduction in Schirmer values may not necessarily indicate clinically relevant tear film dysfunction. The stability of IOP and CCT observed in the present study may reflect the effectiveness of ocular homeostatic mechanisms during moderate exercise. Similar stability of these parameters has been reported in clinically healthy horses, suggesting that moderate physiological challenges may not be sufficient to induce measurable alterations in ocular status (Aragona et al. 2022; Zisopoulou et al. 2023). The reduction in TS observed after exercise may reflect transient physiological adjustments associated with exercise. However, tear film function depends not only on tear volume but also on physicochemical properties, including osmolality and electrolyte composition, which contribute to ocular surface stability in healthy horses (Best et al. 2015). Nevertheless, post‐exercise TS values remained within physiological limits, suggesting that the observed decrease was unlikely to be clinically significant.

The oxidative profile observed in tear fluid, characterised by increased antioxidant enzyme activities and reduced lipid peroxidation, indicates a local biochemical response to exercise‐related oxidative challenge. Notably, a comparable antioxidant‐dominant shift was simultaneously detected in saliva following exercise, suggesting that distinct non‐invasive biological fluids may reflect coordinated oxidative adaptations to physical effort. Exercise‐related modulation of salivary oxidative biomarkers has previously been reported in horses, supporting the responsiveness of non‐invasive matrices to acute physiological stress associated with exercise (Contreras‐Aguilar et al. 2021). In contrast to saliva, which has been more frequently investigated in this context, tear fluid has primarily been studied under basal conditions, and information on its dynamic oxidative response to physical exercise remains limited. The present findings therefore extend existing knowledge by demonstrating that tear fluid reflects exercise‐associated oxidative adaptations in horses. The responsiveness of tear fluid to exercise further supports its potential as a complementary biomatrix in horses.

Recent studies in horses have further supported the usefulness of non‐invasive biological fluids in monitoring exercise‐associated physiological and oxidative responses. Saliva, in particular, has been shown to reflect both stress‐related and exercise‐induced biochemical changes, including antioxidant responses and stress‐related enzymatic activity, under standardised exercise conditions (Contreras‐Aguilar et al. 2021; Hostovská et al. 2024). Similarly, systemic studies have demonstrated that moderate submaximal exercise induces adaptive antioxidant responses rather than purely pro‐oxidant effects, indicating a dynamic redox adjustment rather than overt oxidative damage (Ott et al. 2022). Taken together, these findings support the concept that non‐invasive matrices such as saliva—and potentially tear fluid—can reflect coordinated physiological adaptations to exercise in horses. In this context, tear fluid may represent a promising complementary biomatrix for field‐based monitoring of equine athletes, although further validation across different exercise intensities is required.

Salivary findings are consistent with previous studies showing that saliva is a responsive matrix for detecting physiological changes associated with exercise and training‐related stress in horses (Contreras‐Aguilar et al. 2021; Hostovská et al. 2024). Differences in exercise protocols and sampling time have been reported to influence the magnitude and direction of salivary biomarker responses, which may contribute to the antioxidant‐dominant pattern observed in the present study. In addition, salivary biomarkers have been shown to respond to routine management‐ and handling‐related stressors, even when clinical and haematological variables remain within reference ranges, supporting the sensitivity of saliva for detecting subtle physiological adaptations (Massányi et al. 2023). From a practical perspective, salivary biomarkers may offer a suitable approach for the repeated assessment of exercise‐related oxidative responses under routine training conditions.

This study has several limitations that should be considered when interpreting the findings. First, the exercise protocol was designed to reflect routine submaximal training conditions; therefore, the results may not be directly extrapolated to high‐intensity or competitive exercise. In addition, objective indicators of exercise intensity, including exercise speed, environmental temperature, humidity and percentage of maximal heart rate, were not recorded and therefore could not be evaluated. Second, oxidative stress biomarkers were assessed using saliva and tear fluid, which, although practical and minimally invasive, may be influenced by sampling variability and may not fully reflect systemic oxidative status. In addition, repeatability analyses for these sampling methods were not performed. Furthermore, assay validation for oxidative stress biomarkers in equine tear fluid was not specifically evaluated. In addition, correlation analyses between saliva and tear fluid biomarkers were not performed. Such analyses may have provided further insight into the extent to which both matrices reflect coordinated physiological responses to exercise. Third, the absence of repeated post‐exercise measurements limits evaluation of recovery dynamics. Finally, although a wide age range and both sexes were included to reflect a real‐world horse population, age‐, sex‐ and training status–related influences on ocular parameters and oxidative stress biomarkers cannot be excluded. Because the study was not specifically designed or statistically powered to evaluate these effects, subgroup analyses were not performed. Consequently, differences in age, sex and training history may have contributed to inter‐individual variability and may have influenced the comparability of exercise responses among horses. In addition, because multiple outcome variables were evaluated, the possibility of Type I error cannot be completely excluded despite the hypothesis‐driven nature of the analyses.

## 5 Conclusions

Current findings indicate that routine submaximal exercise can be monitored through non‐invasive assessment of saliva and tear fluid, providing practical insight into exercise‐related oxidative responses in horses. The use of saliva and tear fluid sampling may therefore represent a feasible supportive approach for non‐invasive monitoring of exercise‐associated stress responses in equine practice. The preservation of major ocular parameters suggests that standard training regimens are unlikely to compromise ocular integrity, although subtle changes in TS may be relevant in susceptible individuals. Future investigations incorporating different exercise intensities, extended recovery periods and horses with ocular surface disorders are needed to further clarify the diagnostic and clinical utility of these biomarkers in equine practice.

## Author Contributions

**Ferda Turgut**: investigation, conceptualisation, writing – original draft, methodology, validation, visualisation, data curation, formal analysis. **Mumin Gokhan Senocak**: investigation, writing – review and editing, methodology, formal analysis. **Omer Tarik Orhun**: investigation, conceptualisation, methodology, writing – review and editing. **Betul Apaydin Yildirim**: conceptualisation, investigation, visualisation, validation, methodology. **Yakup Kocaman**: investigation, writing – review and editing, formal analysis, methodology. **Taner Arslan**: investigation, methodology, formal analysis. **Vildan Aslan Canatan**: investigation, methodology, formal analysis. **Latif Emrah Yanmaz**: writing – review and editing, visualisation, methodology, validation, investigation, conceptualisation, formal analysis, supervision.

## Funding

The authors have nothing to report.

## Ethics Statement

Ethical approval for this study was obtained from the Atatürk University Animal Experiments Local Ethics Committee (Approval No. 2025/14‐343).

## Conflicts of Interest

The authors declare no conflicts of interest.

## Data Availability

Data will be made available on request.
